# Genetic control of resistance to salmonellosis and to *Salmonella *carrier-state in fowl: a review

**DOI:** 10.1186/1297-9686-42-11

**Published:** 2010-04-29

**Authors:** Fanny Calenge, Pete Kaiser, Alain Vignal, Catherine Beaumont

**Affiliations:** 1 INRA, UR83 Unité de Recherches Avicoles (URA), 37380 Nouzilly, France; 2 Institute for Animal Health, Compton, Berkshire RG20 7NN, UK; 3 INRA, UMR0444 Laboratoire de Génétique Cellulaire (LGC), 31326 Auzeville, France; 4 Roslin Institute and R(D)SVS, University of Edinburgh, Midlothian EH25 9RG, UK

## Abstract

Salmonellosis is a frequent disease in poultry stocks, caused by several serotypes of the bacterial species *Salmonella enterica *and sometimes transmitted to humans through the consumption of contaminated meat or eggs. Symptom-free carriers of the bacteria contribute greatly to the propagation of the disease in poultry stocks. So far, several candidate genes and quantitative trait loci (QTL) for resistance to carrier state or to acute disease have been identified using artificial infection of *S. enterica *serovar Enteritidis or *S. enterica *serovar Typhimurium strains in diverse genetic backgrounds, with several different infection procedures and phenotypic assessment protocols. This diversity in experimental conditions has led to a complex sum of results, but allows a more complete description of the disease. Comparisons among studies show that genes controlling resistance to *Salmonella *differ according to the chicken line studied, the trait assessed and the chicken's age. The loci identified are located on 25 of the 38 chicken autosomal chromosomes. Some of these loci are clustered in several genomic regions, indicating the possibility of a common genetic control for different models. In particular, the genomic regions carrying the candidate genes *TLR4 *and *SLC11A1*, the Major Histocompatibility Complex (MHC) and the QTL *SAL1 *are interesting for more in-depth studies. This article reviews the main *Salmonella *infection models and chicken lines studied under a historical perspective and then the candidate genes and QTL identified so far.

## Background

Salmonellosis is a zoonotic disease caused by the Gram-negative enteric bacterium *Salmonella*. More than 2500 serotypes have been described, mostly belonging to the species *S. enterica *[1]. Some *Salmonella *serotypes can infect a broad range of domestic animals including poultry, sheep, cattle and pigs and cause symptoms of varying severity ranging from mild gastro-enteritis to death. Some of these serotypes, such as *S. *Typhimurium and *S. *Enteritidis, can infect humans. Other serotypes are host-specific, infecting a single species and generally causing severe, typhoid-like symptoms sometimes leading to death (for instance, *S. *Gallinarum and *S. *Pullorum in poultry). These serotypes can be responsible for disease outbreaks leading to severe economic losses.

Prophylactic measures, vaccination and use of antibiotics are insufficient to eradicate salmonellosis in poultry stocks, whatever the serotype involved. In this context, selection of more resistant chickens can be considered as an alternative solution to decrease occurrence of the disease. The first selection experiments at the beginning of the 20^th ^century aimed to decrease disease occurrence in poultry production systems, which was mainly caused by *S. *Pullorum and *S. *Gallinarum. As food safety became an important concern and these host-specific serotypes were better controlled, the interest of researchers and breeders extended towards decreasing food contamination, mainly due to the serotypes Enteritidis and Typhimurium. *S. *Enteritidis alone, which infects the eggs of contaminated hens, is responsible for one third of the human food poisoning cases in France [2] and of about 15% in the UK in 2007 http://www.defra.gov.uk/foodfarm/farmanimal/diseases/atoz/zoonoses/reports.htm. It does not cause severe symptoms in poultry, but the eggs and meat of infected animals can become a reservoir of infection for the human consumer. In particular, asymptomatic carriers have a major role in *Salmonella *propagation in poultry and hence in food contamination, since they cannot be easily identified and isolated. This is the reason why today resistance to carrier-state ability, and not only to general salmonellosis resistance, is taken into account by some breeders and researchers. Simulation studies demonstrate the usefulness of rearing animals more resistant to carrier state in the prevention of disease propagation in poultry, in synergy with vaccination [3].

Experiments for the selection of genetically resistant animals can be traced back as early as the 1930's [4,5] and the first step was the demonstration that distinct disease resistances or susceptibilities exist between different lines or breeds of chicken. The second step consisted in evaluating the heritability of disease resistance-related traits, which confirmed that the observed variability among lines had a genetic origin [6-8]. Next, genomic regions responsible for the observed genetic variability were identified, which provided a better understanding of the mechanisms involved in resistance and should theoretically lead to marker-assisted selection (MAS). MAS can potentially accelerate the selection process, and prevent infection of animals. To date, two different approaches have been used successfully to unravel the genetic control of disease resistance variability, i.e. (1) candidate gene approaches with *a priori *knowledge of the genes potentially involved (for instance, [9-11]) and (2) quantitative approaches through quantitative trait locus (QTL) analyses, which have been conducted since the development of molecular markers in the 1990's [12-15]. A final step towards obtaining more resistant animals is selection itself, with or without the contribution of molecular markers. The feasibility of selection for increased resistance to *S. *Enteritidis carrier-state has been demonstrated [16]. Nevertheless, molecular markers still have to be included in the selection process, in order to take advantage of the recent knowledge acquired on genetic resistance mechanisms.

In this article, we review the literature on studies aimed at identifying the genes responsible for variable resistance to salmonellosis in chicken. The article is organised as follows: (1) the different *Salmonella *infection models, (2) the genetic resources used, (3) the candidate gene approaches, (4) the QTL analyses conducted and (5) the co-localisations occurring between candidate genes and QTL.

### 1. The *Salmonella *infection models: a historical perspective

Many different *Salmonella *infection protocols are described in the literature. Here, we focus on the protocols that have been used for genetic studies. Many factors have to be taken into account to assess *Salmonella *resistance i.e. infectious doses, *Salmonella *serotypes and strains, route and age of infection, delay between infection and phenotypic observations, and the animal rearing conditions. In addition, different parameters can be measured: survival rate, lethal dose leading to 50% of dead animals (LD50), internal organ contamination, presence/absence of *Salmonella*, *Salmonella *count, etc. The main infection models used to identify genes for resistance to *Salmonella *are summarized in Table 1.

**Table 1:** Infection models used in published studies of the genetic control of resistance to *Salmonella *in fowl

**Locus type**^**1**^	Infection route	**Age**^**2**^	**Time**^**3**^ (pi)	**Trait**^**4**^	Cross type	**Parental lines**^**5**^	**Ref**^**6**^
MSAT	subcuta-neaous	10 d	10 d	ABR to SE vaccine	F2+BC	(low × high) ABR divergent inbred lines	[15]
MSAT CG	subcuta-neaous	10 d	21 d	ABR to SE vaccine	F1	Broiler outbred male × 3 inbred lines (2 MHC-congenic WL + Fay)	[12] [64]
QTL	oral	1 w	4/5 w	CSWB counts/caecal load	F2	(N × 6_1_) × (N × 6_1_) layer inbred lines	[14]
QTL	oral	6 w	2 w	CSWB counts/caecal load	BC	(N × 6_1_) × 6_1 _layer inbred lines	[14]
QTL	oral	2 w	5 d	splenic load	BC	(6_1 _× 15I) × 6_1 _layer inbred lines	[13]
CG	subcuta-neaous	10 d	11 d	ABR to SE vaccine	F2	(Fay × WL) × (Fay × WL)	[66]
CG	intra-oesophageal	10 d	21 d	ABR to SE vaccine	F1	Broiler outbred male × 3 inbred lines (2 MHC-congenic WL + Fay)	[61-63]
CG	intra-oesophageal	1 d	7/8 d	spleen and caecal loads	F8	AIL (Broiler × Fay) × AIL (Broiler × inbred WL)	[59]
CG	intravenous	13 w	3 d	spleen and liver loads	F1	Egg-type commercial crosses	[7]
CG	oral	peak of lay	4 w	spleen load; number of contaminated organs	F1	Egg-type commercial crosses	[9]
CG	intra-muscular	1 d	death or 2 w	survival rate	BC	(WlxC) × C	[10]
CG	intra-muscular	1 d	death or 2 w	survival rate	F0	Inbred WL lines	[54]
CG	intra-oesophageal	1 d	6/7 d	caecal and spleen loads	F1	Broiler outbred male × 3 inbred lines (2 MHC-congenic WL + Fay)	[55,61-63,78]
CG	intra-oesophageal	1 d	6 d	caecal and spleen loads	F8	(Broiler × Fay) × AIL (Broiler × inbred WL)	[60]
CG	oral	3 w	7 d	caecal load	F0	5 groups of meat type chicken	[11]

^1 ^CG: candidate gene, MSAT: microsatellite

^2 ^Animal age at infection or injection; d: day; w: week

^3 ^Assessment time post inoculation (pi)

^4^ABR: antibody response; CSW: cloacal swab; SE: *Salmonella *Enteritidis

^5 ^AIL: advanced intercross lines; Fay: Fayoumi; WL: White Leghorn

^6 ^Reference

At the beginning of the 20^th ^century, the breeder's main objective was to reduce mortality in industrial poultry stocks. For practical reasons, *Salmonella *resistance assessment was carried out on young chicks (1 day to 2 weeks). Chicks are more susceptible to salmonellosis than adults, so that discrimination among animals was evaluated via their survival rates. Chicks were infected with a high dose of the serotypes that were known to cause the most severe symptoms in infected chicken, i.e. *S. *Pullorum, *S. *Gallinarum and *S. *Typhimurium [4,5,17-20]. Some studies also reported infection of hens at peak of lay [21], because the possibility of vertical transmission of bacteria to eggs was already a concern. Different infection routes were used according to the study: oral [19-21], intraperitoneal [4], or subcutaneous [17]. With the improvement of alternative disease control practices, such as chemotherapy, competitive exclusion, prophylactic measures, use of antibiotics and vaccination, disease outbreaks in poultry stocks were reduced and the interest in selection for *Salmonella *resistance decreased.

In the 1980s, the number of human food poisoning outbreaks increased, mainly due to *S. *Enteritidis, which renewed the interest to select more resistant animals. Several studies aimed at comparing the effects of different serotypes on mortality rates, and of the route of inoculation (intramuscular or oral) were carried out on day-old chicks [22-24]. A few studies assessed the carrier state of chickens infected with *S. *Enteritidis, since symptom-less carriers are the main cause of disease propagation in poultry. In such studies, the persistence of bacteria in infected chickens has to be assessed several weeks post-infection. Guillot et al. [25] infected day-old chicks with high doses (orally or intra-muscularly) but followed the persistence of bacteria in several internal organs, in addition to measuring mortality. Duchet-Suchaux et al. [26,27] developed a model in which one week-old chicks were orally infected with a smaller dose of bacteria, thus preventing mortality and disease symptoms, in order to observe the persistence of bacteria in different organs several weeks after infection. The carrier-state in adult chickens has been less well studied. Protais et al. [28] and Lindell et al. [29] orally infected adult hens at peak of lay and followed the persistence of bacteria in different organs.

In the above studies, *Salmonella *resistance was assessed by observing survival rates or quantities or presence/absence of bacteria in different organs. In more recent studies, indirect, linked parameters have been used to characterise *Salmonella *resistance: innate or adaptive immunity-related traits [30-32], antibody response after a *S*. Enteritidis vaccine [12,15], or gene expression by genome-wide, microarray analyses [33-35] or more targeted studies focusing on one or several genes [36-41]. Observation of these traits contributes to a better understanding of the immunological and transcriptional mechanisms involved in resistance differences between lines.

### 2. Comparing *Salmonella *resistance levels between chicken lines

The first step towards the identification of resistance genes is to choose and mate parental lines that differ in *Salmonella *resistance levels. Phenotypic variation is very high in poultry. For research purposes, inbred lines derived from selected breeds are the material of choice because of their higher rate of homozygosity and their relationship to actual commercial breeds. The first published studies at the beginning of the 20^th ^century reported comparisons of different layer lines, i.e. mainly White Leghorn and Rhode Island Red lines [4,5,17-21]. Most of these studies mention the greater resistance of the Rhode Island Red compared to the White Leghorn lines. The following studies used inbred or partially inbred lines generated from commercial layer or broiler lines. Mortalities after *S. *Typhimurium or *S. *Enteritidis infection of the inbred lines N, C, 15I, Wl, 6_1_, 7_2 _and 0, all derived from White Leghorn layer lines, have been compared [22-24,42]. Lines C, 7_2 _and 15I were always more susceptible, whereas lines N, 6_1 _and Wl were always more resistant to infection. This line ranking was identical whatever the serotype used. Mortality and persistence of bacteria in internal organs were compared in the experimental White Leghorn inbred lines B13 and Y11, in the meat-type experimental line Y11, and in a commercial line (L2) [25-27]. Some studies used lines which were especially selected to study disease resistance: for instance, divergent lines for low/high antibody response [25].

The effects of genetic differences in resistance to *Salmonella *can be investigated by studying traits related to the immune response on different chicken lines. Heterophil functionality has been measured in several commercial lines of birds differing in their resistance to *S. *Enteritidis [43-45]. Crop immune response has been measured in eight commercial layer hens and White Leghorn chickens [32]. Some studies report genetic differences for the antibody response to *S*. Enteritidis [15,46,47]. Similarly, many studies report gene expression differences between different chicken lines after artificial infection, identified by genome-wide, microarray analyses [33-35] or more targeted studies focusing on one or several genes [36-41]. Other studies used lines selected for other traits (such as growth rate or feed conversion efficiency [33,48]), which makes it possible to investigate the interaction between the main trait under study and *Salmonella *resistance.

### 3. Candidate gene approaches

A candidate gene approach requires *a priori *knowledge of the genes potentially involved in *Salmonella *resistance. The first candidate gene tested in chicken was chosen on the basis of genetic studies carried out in mice infected by *S. *Typhimurium. This gene, *NRAMP1 *(natural resistance-associated macrophage protein, now *SLC11A1*), has been identified on mouse chromosome 1, under the name Ity (Immunity to Typhimurium), after mice strains were classified into two categories: resistant vs. susceptible, as reviewed in [49]. The identity of Ity with two other genes, Bcg and Lsh, involved in resistance to, respectively, *Mycobacterium bovis *and *Leishmania donovani*, was demonstrated after the positional cloning of a unique gene, *NRAMP1 *[50]. *NRAMP1 *has since been described as a member of a solute carrier gene family and hence renamed *SLC11A1*. Physiological and functional studies support the role of *SLC11A1 *in the control of the intracellular replication of parasites in phagosomes. A homologue of *NRAMP1 *has been mapped on chicken chromosome 7 [51,52] and cloned subsequently [53]. Another major gene, *TLR4 *(Toll-like receptor 4), previously named *Lps*, belongs to a family of innate immune system receptors (Toll-like receptors) and is involved in the recognition of LPS (lipo-polysaccharide) from Gram-negative bacteria. *Lps *was mapped to mouse chromosome 4 after analysis of mouse strain C3H/HeJ which has both a hypo-responsiveness to LPS motifs and a higher susceptibility to *S. *Typhimurium. Positional cloning of *Lps *led to the identification of *TLR4 *as a positional candidate. The chicken homologue of *TLR4 *has been mapped to micro-chromosome 17 and cloned [54].

Several studies have attempted to determine whether *SLC11A1 *and *TLR4 *are involved in resistance variation to *S. *Typhimurium and *S. *Enteritidis. The survival rate of young chicks derived from a backcross between lines W1 and C and infected intra-muscularly one day post-hatch with *S. *Typhimurium was linked to *SLC11A1 *and *TLR4*, which, together, explained up to 33% of the differential resistance to infection [10,54]. This effect was observed only during the first seven days post-infection. An effect of *SLC11A1 *on the early stages of systemic *Salmonella *infection using day-old chicks was confirmed in five groups of meat-type chickens [11] and in F1 progenies derived from crosses between a broiler line and Fayoumi or MHC-congenic lines [55,56].

Since human *Salmonella *infection is mainly due to the consumption of eggs or meat from adult chickens, commercial egg-type chickens intravenously infected with *S. *Enteritidis have also been studied but at 13 weeks instead of at a young age [7]. Similarly, it has been demonstrated that a marker closely linked to *SLC11A1 *displayed a within-sire effect on liver and spleen load assessed early (three days post-infection), which confirms the possible involvement of *SLC11A1 *early in the process of systemic infection in these chicken lines, although infection occurred at an older age. Following bacterial contamination several weeks after infection is the only way of studying the *Salmonella *carrier-state. Thus, the potential role of *SLC11A1 *in later stages of the infection was demonstrated, firstly in mice inoculated with *S. *Enteritidis at 8-10 weeks with spleen bacterial counts, 42 days post-infection [57]. Interestingly, it seems that different *SLC11A1 *alleles were involved in early vs. late resistance. The same allele may be involved both in resistance to colonisation in early stages of the infection and in a high excretion rate in later stages. Similarly, an effect of *SLC11A1 *on spleen contamination was then demonstrated in chicken lines orally inoculated at peak of lay and slaughtered four weeks later [9], while in the same study the role of *TLR4*, although suspected, was not confirmed. More recently, the effect of the *SLC11A1 *locus was found significantly associated with carrier-state resistance variations in divergent chick lines [58].

In addition to these two genes, many genes related to immune response in chicken have been tested for their association with caecal or splenic load after *S. *Enteritidis challenge of one-day- to three-week-old chicks (Table 2; [11,54,55,59-63]). Other studies have focused on the antibody response to *S. *Enteritidis vaccination [62-66]. These studies exploit either polymorphisms found in the gene itself (mainly SNP) or closely associated genetic markers. Most of these genes have been tested in progenies derived from crosses between White Leghorn MHC-congenic inbred lines and inbred Fayoumi lines. Such crosses between genetically distant parental lines are an efficient way of maximising genetic variation. However, genes identified in this way may be fixed in other populations, so that their interest for selection purposes needs to be validated.

**Table 2:** Physical and genetic positions of published loci for resistance to *Salmonella *in fowl.

**Chr**^**1**^	**Locus type**^**2**^	Locus name	**Trait**^**3**^	**Position**^**4**^**cM**	Mb	Ref
**1**	MSAT	ADL0160	ABR to SE vaccine	33	5.93	[15]

	QTL	-	CSWB counts (SE)	85	33.57	[14]

	QTL	-	CSWB counts (ST)	207	68.52	[14]

	MSAT	ADL0020	ABR to SE vaccine Splenic and caecal loads (SE)	286	94.16	[12] [78]

	CG	*CD28*	Caecal load; ABR to SE vaccine	-	113.90	[62]

	MSAT	ADL0198	ABR to SE vaccine Splenic and caecal load (SE)	458	171.74	[12] [78]

	CG	*IAP1*	caecal load (SE) Splenic load (SE)	-	186.92	[11] [55]

**2**	QTL	-	CSWB counts (SE)	87	26.93	[14]

	CG	*MD-2*	Splenic load (SE)	-	122.83	[62]

	MSAT	MCW0051	ABR to SE vaccine	358	129.15	[15]

**3**	MSAT	MCW0083	ABR to SE vaccine	51	13.99	[15]

	MSAT	MCW0024	ABR to SE vaccine	237	-	[15]

	CG	*TGF-β4*	Caecal load (SE)	-	18.29	[11]

	CG	*TGF-β2*	Caecal load (SE) ABR to SE	-	20.54	[11] [66]

	CG	Gal13	Caecal load (SE)	-	110.20	[60]

	CG	Gal12	Caecal load (SE)	-	110.21	[60]

	CG	Gal11	Caecal load (SE)	-	110.21	[60]

	CG	Gal7	ABR to SE vaccine	-	110.25	[64]

	CG	Gal3	ABR to SE vaccine Caecal load (SE)	-	110.26	[60] [64]

	CG	Gal5	Spleen load (SE)	-	110.27	[60]

**4**	CG	*TRAIL*	Spleen and caecal load (SE)	-	9.67	[63]

	CG	*IL-2*	Caecal load (SE)	-	55.26	[11]

**5**	QTL	-	CSWB counts (ST)	100	36.10	[14]

	QTL	-	CSWB counts (SE)	111	39.28	[14]

	QTL QTL	*SAL1* *SAL1*	Splenic load (ST) Splenic load (ST)	157 -	53.24 54.00-54.80	[13] [74]

	MSAT	ADL0298	ABR to SE vaccine Splenic and caecal load (SE)	198	60.23	[12] [78]

	CG	*TGF-β3*	Caecal load (SE)	-	40.87	[63]

**6**	MSAT	ADL0138	ABR to SE vaccine Splenic and caecal load (SE)	56	10.09	[12] [78]

	CG	*PSAP*	Splenic and caecal loads (SE)	-	13.02	[11] [55]

**7**	CG	*SLC11A1*	Survival rate (ST) Splenic and liver loads (SE) Splenic load (SE) Splenic load (SE); number of contaminated organs Splenic load (SE); ABR to SE vaccine Caecal load (SE)	80	23.91	[10] [7] [55] [9] [61] [79]

**8**	MSAT	ADL301	ABR to SE vaccine	80 EL	25.10	[15]

**11**	QTL	-	Caecal load (SE); CSWB counts	18	3.66	[14]

**15**	CG	*IGL*	Caecal load ABR to SE vaccine	-	8.17	[11] [63]

**16**	QTL	-	Caecal load	2	0.10	[14]

	CG	*MHC1*	Splenic load (SE)	-	-	[55]

	CG	*MHC *class I α1 domain	ABR to SE vaccine	-	0.04	[80]

	CG	*MHC *class I α2 domain	ABR to SE vaccine		0.04	[80]

	CG	*MHC *class I β1 domain	ABR to SE vaccine		0.06	[80]

**17**	CG	*TLR4*	Survival rate (ST) Survival rate (ST) Number of contaminated organs	-	4.06	[10] [54] [9]

	MSAT	ADL0293	ABR to SE vaccine	26	6.39	[15]

**19**	CG	*CASP1*	Caecal load	-	0.64	[11]

	CG	*iNOS*	Caecal load	-	9.17	[11]

**26**	CG	*PIGR*	Splenic and caecal loads	-	0.00	[59]

	CG	*MAPKAPK12*	Splenic and caecal loads	-	2.35	[59]

	CG	*IL10*	Splenic and caecal loads	-	2.37	[59]

**28**	MSAT	LEI0135	ABR to SE vaccine	0	0.18	[15]

^1^Chromosome

^2^CG: candidate gene; MSAT: microsatellite

^3^ABR: antibody response; CSWB: cloacal swabs; ST: *S. *Typhimurium; SE: *S. *Enteritidis

^4^Physical positions were obtained by searching the Ensembl Genome Browser http://www.ensembl.org/index.html with the original Accession Number given by the authors. QTL positions were calculated according to physical positions of flanking molecular marker.

Many genes have been identified in gene expression studies. Most of them are probably not directly responsible for the actual genetic variation between these lines, but they remain functional candidates until they are tested for their role in genetic variation. Genome-wide microarray studies have led to the identification of genes differentially expressed between different chicken lines infected with *S*. Enteritidis [33-35,67] or before/after infection with *S. *Enteritidis [31,38,68]. Other genes have been more specifically studied, such as for instance genes coding for cytokines [69,70], Toll-like receptors [37,71,72] or innate immune response genes [39].

### 4. QTL analyses

Targeted candidate gene analyses have very rarely led to the complete unravelling of the heritable part of phenotypic variations. On the contrary, QTL analyses are designed to encompass the greatest part possible of the observed variability, with the inconvenience that the genomic regions identified are anonymous and often contain several hundred genes. Until now, few QTL studies have been carried out to identify genes for acute resistance or resistance to carrier-state in chicken (Table 1). The first QTL study of *Salmonella *resistance analysed data from a back-cross progeny produced from White Leghorn inbred lines ((6_1 _× 15I) × 15I) and infected at two weeks of age with *S. *Typhimurium [13]. A major QTL controlling spleen bacterial load was identified on chromosome 5 and named *SAL1*. *SAL1 *was shown to be involved in bacterial clearance by macrophages [73]. Using a 6^th ^generation backcross mapping population and high density SNP panels, the *SAL1 *locus was confirmed and its localisation was refined at a position between 54.0 and 54.8 Mb on the long arm of chromosome 5 [74]. This region spans 14 genes, including two very striking functional candidates: CD27-binding protein (Siva) and the RAC-alpha serine/threonine protein kinase homolog, AKT1 (protein kinase B, PKB). AKT1 is involved in cellular survival pathways, primarily by inhibiting apoptotic processes. Survival factors can suppress apoptosis in a transcription-independent manner by activating AKT1, which then phosphorylates and inactivates components of the apoptotic machinery. AKT1 can also activate NF-κB by regulating IκB kinase (IKK), resulting in transcription of pro-survival genes and stimulation of pro-inflammatory responses [75]. Hijacking of this pathway by the *Salmonella *effector protein SopB provides support for *AKT *as a plausible candidate gene for bacterial proliferation and its association with the susceptibility/resistance status of the host.

**Figure 1:**
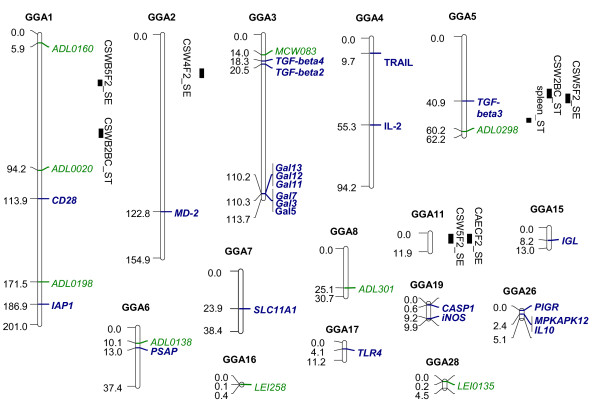
**Physical map of published loci for resistance to *Salmonella *in fowl**. Mapchart 2.1 software was used to draw this map [77]. Positions are indicated in Mb. QTL positions are indicated by plain black boxes to the right of chromosomes; their length was calculated to cover 20 cM centered on the QTL likelihood peak. ABR: antibody response; SE: *Salmonella *Enteritidis; ST: *Salmonella *Typhimurium.

QTL for carrier-state resistance have been identified in one back-cross and one F2 progeny, both derived from the White Leghorn inbred lines 6_1 _and N, infected at one week post-infection with either *S. *Typhiumurium (BC) or *S. *Enteritidis (F2) and assessed for their caecal and caecal lumen content bacterial loads two to six weeks later [14]. One genome-wise significant QTL on chromosome 2 and five chromosome-wise significant QTL on chromosomes 1, 5, 11 and 16 were identified (Table 2; Figure 1). Some QTL were specific to one of the two progenies studied (BC vs. F2), which can be attributed to differences in the progeny types, the serotypes used for infection, or the times of infection and phenotypic assessments. Different QTL were found for the caecal bacterial load and the caecal lumen bacterial load. Two of these QTL, on chromosomes 2 and 16, have recently been confirmed in a more targeted analysis of the same progeny [58]. Interestingly, two QTL on chromosomes 1 and 16 were validated in a completely different genetic background, i.e. lines derived from commercial chicken lines [58]. Thus, genetic studies conducted on experimental lines can be of potential interest for marker-assisted selection in commercial lines. Furthermore, two different sets of QTL and candidate genes have been confirmed in adult chickens and in chicks derived from the same commercial line, which strengthens the hypothesis of a genetic control of *Salmonella *carrier-state differing according to chicken's age previously formulated [16].

Other studies have more specifically focused on the antibody response to *S. *Enteritidis vaccination. Associations were found between microsatellite markers and traits related to the antibody response to *S. *Enteritidis vaccination, from data obtained respectively from BC and F2 progenies derived from inbred lines selected for high/low antibody response and from F1 families derived from crosses between a broiler and either MHC-congenic White Leghorn lines or the Fayoumi line [15,12]. Nevertheless, the significant microsatellites identified were not located in the same genomic regions. This could be due to genetic differences between the parental lines studied, but also to differences in the experimental conditions (Table 1). The time of assessment and possibly the vaccine used were different and may have influenced the outcome of infection.

### 5. Genomic organisation of *Salmonella *resistance loci

The different candidate genes, QTL and microsatellites significantly linked to *Salmonella *resistance are shown in Figure 1. These loci are located on 16 of the 38 autosomal chromosomes of the chicken genome. Microchromosomes are poorly represented, due to the lack of genetic markers and genome sequences in these regions. Genomic co-localisations reveal a possible common genetic background explaining variations for resistance under different experimental conditions. Genetic or physical co-localisations indicate the possibility of the co-localised loci being identical, although the possibility of close physical linkage between adjacent genes should obviously never be discarded. Three types of genetic co-localisations can be observed between the candidate genes and the *Salmonella *resistance QTL mentioned above. First, several co-localisations occur between QTL for antibody response-related traits [15] and candidate immune-response genes: two on chromosome 1, one on chromosome 3, and one on chromosome 6. Before the immunity-related genes can be considered as relevant candidates for the co-localising QTL, ideally they should be tested in the same conditions as the QTL with which they co-localise, i.e. in particular with the same phenotypic trait, in the same or similar progeny, using the same *Salmonella *serotype under the same infection or vaccination model. The absence of other potentially relevant candidates should also be verified in the QTL confidence intervals. Secondly, a cluster can be observed on chromosome 5, including two QTL for resistance to *S. *Enteritidis and *S. *Typhimurium [14], one QTL for the antibody response to *S. *Enteritidis vaccination [12], the QTL *SAL1 *and the *TGF-β3 *gene. It is theoretically possible that all these QTL are actually the same gene, although the refined *SAL1 *locus does not include *TGF-β3 *[74]. The molecular cloning of *SAL1*, which is so far the QTL with the most important effect identified, would solve this question. Finally, a co-localisation involves the MHC on micro-chromosome 16 and a *S. *Enteritidis carrier-state QTL [14]. Due to the high density of immunity-related genes and to the poor recombination rate observed on this chromosome, identifying which gene is the causal gene at this QTL will not be easy.

## Conclusion

Several candidate genes and QTL have been successfully identified as having roles in phenotypic variations related to *Salmonella *resistance. Despite the many differences in infection models and genetic materials used and in traits assessed, which make the comparison of these loci somewhat speculative, great progress has been achieved in the last few years to understand the genetic control of resistance to *Salmonella*. The diverse experimental conditions used lead to a complex sum of results, but allow a more complete description of the disease. Resistance to salmonellosis and *Salmonella *carrier state varies according to the chicken line under study, the chicken's age, and the trait assessed, and probably many other parameters which have not been studied yet. Comparisons of the different models used raise many questions. In particular, the genetic differences between acute and carrier-state resistance and the influence of the chicken's age on resistance are interesting theoretical issues which still need to be investigated thoroughly before selection is considered. The genomic regions carrying the candidate genes *TLR4 *and *SLC11A1*, the Major Histocompatibility Complex (MHC) and the QTL *SAL1*, identified using several infection models, are interesting candidates for more in-depth studies.

With the development of high-throughput technologies such as microarray expression analyses and RNA-seq [76], new-generation sequencing (NGS) technologies and high density SNP genotyping, a huge quantity of differentially expressed candidate genes and polymorphisms is already available, which should speed up the unravelling of the *Salmonella *resistance genetic mechanisms. The most limiting factors are and will clearly remain the frequent and inevitable lack of precision and reliability of phenotypic assessments and the poor density of genetic recombinations in the progenies under study, which both limit the precision of QTL localisation and fine-mapping. Another limiting step resides in the choice of the relevant differentially expressed genes to be tested for their involvement in genetic variation.

All these studies will no doubt lead to a large number of genes or genome regions involved in *Salmonella *resistance variation and extend our theoretical knowledge of the genetic control of this disease. However, for practical applications, i.e. to implement marker assisted selection in commercial populations, it will be important to identify which of these genes are the most important. The answer will vary according to the chicken population under study and the selection criteria used, which clearly is an obstacle to practical application. Genomic selection may soon settle this matter by the direct selection of resistance-related traits in populations under selection.

This new knowledge of the genetic architecture of *Salmonella *resistance in fowl, in addition to genomic selection, could soon lead to the selection of more resistant animals. Combined with other measures, it should contribute in reducing the spread of the disease in commercial flocks.

## Competing interests

The authors declare that they have no competing interests.

## Authors' contributions

FC wrote the manuscript. PK contributed to the chapters related to candidate genes and genomic approaches. AV contributed to the chapters related to genomics approaches and QTL detection. CB contributed to the chapters related to genetic selection and infection models. All authors read and approved the final manuscript

## List of abbreviations used

MAS: Marker Assisted Selection; MHC: Major Histocompatibility Complex; QTL: Quantitative Trait Locus; SNP: Single Nucleotide Polymorphism.
